# Longitudinal Outcomes of Patients with Aortic Stenosis Stratified by Sex: An Asian Perspective

**DOI:** 10.3390/jcdd12010032

**Published:** 2025-01-19

**Authors:** Joy Y. S. Ong, Aloysius S. T. Leow, Chun Yi Ng, Poay Huan Loh, Swee Chye Quek, William K. F. Kong, Tiong Cheng Yeo, Ching Hui Sia, Kian Keong Poh

**Affiliations:** 1 Department of Cardiology, National University Heart Centre Singapore, 5 Lower Kent Ridge Road, Singapore 119074, Singapore; joy.ong@mohh.com.sg (J.Y.S.O.); mdcv538@visitor.nus.edu.sg (C.Y.N.); poay_huan_loh@nuhs.edu.sg (P.H.L.); william_kong@nuhs.edu.sg (W.K.F.K.); tiong_cheng_yeo@nuhs.edu.sg (T.C.Y.); kian_keong_poh@nuhs.edu.sg (K.K.P.); 2 Department of Medicine, National University Hospital, Level 10, NUHS Tower Block 1 Kent Ridge Road, Singapore 119228, Singapore; aloysius.leow@mohh.com.sg; 3 Department of Medicine, Yong Loo Lin School of Medicine, National University of Singapore Level 11, NUHS Tower Block 1E Kent Ridge Road, Singapore 119228, Singapore; paeqsc@nus.edu.sg

**Keywords:** aortic stenosis, sex differences, Asian population, clinical outcomes

## Abstract

Background: Severe aortic stenosis (AS) stratified by sex has been increasingly studied in the European population. Sex-specific outcomes in Asian patients with AS remain poorly defined. Hence, we aimed to study the clinical characteristics and impact of sex in moderate-to-severe AS, undergoing both invasive and conservative interventions in an Asian cohort over 10 years. Methods: Consecutive data with echocardiographic diagnoses of AS were stratified according to gender in a tertiary academic center between 2011 and 2021. Demographics, comorbidities, and clinical outcomes were compared. Results: Seven hundred and three (703) patients were included (56%, *n* = 397 were female). Calcific AS was the dominant etiology in both genders. Females had higher incidences of anemia (*p* < 0.001) and chronic kidney disease (*p* = 0.026); although, females had lower incidences of cardiovascular complications of coronary artery disease (CAD) (*p* = 0.002) and prior acute myocardial infarction (AMI) (*p* = 0.015). Echocardiographically, females had a smaller left ventricular outflow tract diameter (LVOTd) (*p* < 0.001), LV mass (*p* < 0.001), and left ventricle end diastolic volume (LVEDV) (*p* < 0.001). Conversely, the left atrial (LA) area (*p* < 0.001) and volume index (LAVI) (*p* < 0.001) were larger in females. Females had higher average E/e’ (*p* = 0.010) ratios compared to males. The mean follow-up duration between genders was 4.1 ± 3.3 years. Upon univariate analysis, a greater proportion of female AS patients encountered cardiovascular (CV) hospitalization during follow-up (female: 27.5%, *n* = 109 vs. male: 18.3%, *n* = 56; *p* = 0.005) compared to male patients, but there were no significant differences for the outcomes of heart failure (*p* = 0.612), stroke (*p* = 0.664), and all-cause mortality (*p* = 0.827). Fewer females underwent aortic valve (AV) intervention compared to males (21.2% vs. 27.8%, *p* = 0.042), albeit with a longer duration to AV intervention (3.6 years ± 2.4 vs. 2.6 years ± 2.3, *p* = 0.016). In the severe AS cohort, female sex remained an independent predictor for subsequent heart failure (aHR 2.89, 95% CI 1.01–8.29, *p* = 0.048) and CV hospitalization (aHR 20.0, 95% CI 1.19–335, *p* = 0.037) after adjustments for age, ethnicity, body mass index (BMI), comorbidities, left ventricular ejection fraction (LVEF), and AV intervention. Conclusions: There was no difference in heart failure, stroke, and all-cause mortality outcomes between male and female Asian patients with moderate-to-severe AS. However, there were more cardiovascular hospitalizations, with fewer and longer duration to AV intervention in females compared to males in our cohort.

## 1. Introduction

Sex differences in the pathobiology of aortic stenosis (AS) have a profound impact on downstream therapeutics and outcomes. An increasing spotlight has been cast on sex-based differences in severe aortic stenosis (AS) in Europe over the past decade [1,2]. There has been published work on postulated cellular mechanisms, clinical characteristics, and transcatheter aortic valve replacement (TAVR) options between sex with a particular focus on severe AS in Western cohorts [1,2]. Anatomically, females have smaller aortic annuli with increased fibrosis and less calcification of the valve [3]. Physiologically, impaired myocardial perfusion reserve and compensatory left ventricular response to pressure overload leads to more concentric remodeling of the left ventricle in females. There are also elevated incidences of female patients with paradoxical low-flow, low-gradient AS, with fewer rates receiving definitive aortic valve replacement (AVR). European females who underwent AVR had better outcomes than males [3].

While studies have been carried out to outline the sex-based clinical differences in AS in European cohorts, characteristics and the outcomes of sex in Asian patients with AS remain poorly defined. A prior study conducted by Ngiam et al. first described sex-based features and better outcomes of female Asian patients with severe AS who were conservatively managed [4]. However, there remains a knowledge gap in the landscape beyond severe-grade AS patients undergoing valvular interventions.

In our study, we aimed to evaluate key characteristics and clinical outcomes of moderate-to-severe AS undergoing both surgical and transcatheter valvular intervention between male and female patients in an Asian cohort.

## 2. Methods

### 2.1. Study Design and Population

This was a retrospective observational study carried out in a tertiary academic hospital in Singapore. Consecutive patients diagnosed with AS based on an echocardiography registry from September 2011 to December 2021 were included. Patients with concomitant significant valvular diseases were excluded. Ethics approval was obtained from the Domain Specific Review Board (DRSB). In patients with multiple echocardiographic studies during the study period, only the index echocardiography was considered. The grading of the AS severity and evaluation of echocardiographic parameters were carried out in accordance with the European Association of Cardiovascular Imaging (EACVI) and the American Society of Echocardiography (ASE) guidelines [5]. Baseline demographics, relevant clinical characteristics, echocardiographic parameters, laboratory investigations, treatment, and outcomes were obtained from the electronic medical records. Patients were stratified by sex and analyzed, with subgroup analyses performed on moderate and severe AS patients.

### 2.2. Study Endpoints and Statistical Analysis

The primary endpoint studied was all-cause mortality, while the secondary endpoints included aortic valve (AV) intervention, subsequent heart failure, stroke outcome, and hospitalization due to cardiovascular (CV) events.

We presented categorical variables in terms of percentages and frequencies and continuous variables as means ± standard deviations. Categorical variables were compared with the chi-square test, and continuous variables with independent samples *t*-tests. For survival analyses, a Kaplan–Meier estimate was plotted, and the difference was analyzed with the log-rank test. We accounted for the competing risk of all-cause mortality by performing cumulative incidence function estimates for the secondary outcomes of subsequent heart failure, stroke, and CV hospitalization [6]. Time-to-event analyses were performed using the Cox proportional hazards regression model for all-cause mortality and the Fine and Gray competing risks (for all-cause mortality) regression model for aortic valve intervention for severe AS, subsequent heart failure, and CV hospitalization outcomes, which were presented as adjusted hazard ratio (aHR), 95% confidence interval (95% CI), and *p*-value [7]. The variables for the multivariable models were selected a priori based on a background literature review, which adjusted for age, sex, ethnicity, BMI, comorbidities (coronary artery disease [CAD], previous stroke or transient ischemic attack [TIA], chronic kidney disease [CKD], anemia), and left ventricular ejection fraction (LVEF). The presence of aortic valve (AV) intervention was also included for the multivariable models in severe AS patients. The outcomes of stroke were not analyzed in the regression analyses due to the low incidence rates. All *p*-values < 0.05 were considered statistically significant. The statistical analysis was conducted using R Statistical Software (v4.3.1; R Core Team, 2023), Rstudio 12.1 (v2023.12.1; Rstudio Team, 2024) with the following key packages: ggsurvfit (v1.0.0, Sjoberg, 2024) and tidycmprsk (v1.0.0, Sjoberg, 2023).

## 3. Results

A total of 703 AS patients were included in this study, of which 397 were female (56.5%), and 437 were of Chinese ethnicity (62.2%) (Table 1).

There were no significant differences in terms of age (female: 75.1 ± 12.0 years old vs. male: 73.5 ± 12.7; *p* = 0.084). Body surface area (BSA) was smaller in female (1.6 ± 0.2 m^2^) compared to male patients (1.7 ± 0.2 m^2^) (*p* < 0.001), but there was no significant difference in BMI between sex (female: 25.2 ± 5.6 kg/m^2^ vs. male: 24.4 ± 5.1 kg/m^2^; *p* = 0.051).

Fewer female AS patients had cardiovascular risk factors or diseases, such as current (*p* < 0.001) or previous smoking (*p* < 0.001), coronary artery disease (CAD) (*p* = 0.002), previous acute myocardial infarction (AMI) (*p* = 0.015), and chronic obstructive pulmonary disease (COPD) (*p* = 0.015) than male patients, except for chronic kidney disease (CKD) (*p* = 0.026), which was more prevalent in female patients. Hemoglobin levels were lower in female (11.4 ± 2.0 g/dL) compared to male patients (12.4 ± 2.3 g/dL) (*p* < 0.001), and fewer female AS patients were found to be on aspirin (*p* = 0.024). More females had higher grade New York Heart Association (NYHA) Class 4 symptoms (females 16.7%, *n* = 33 vs. male: 6.8%, *n* = 10, *p* = 0.043)

In terms of echocardiographic data, both female (33.3%, *n* = 55) and male (36.0%, *n* = 54) AS patients had a similar proportion of severe AS (*p* = 0.126). There were no significant differences in terms of AS etiology and echocardiographic indices, such as mean pressure gradient (MPG) (*p* = 0.106), peak pressure gradient (PPG) (*p* = 0.364), maximum velocity (Vmax) (*p* = 0.481), and indexed stroke volume (SVi) (*p* = 0.780). Female AS patients had significantly smaller anatomic parameters of left ventricular outflow tract (LVOT) diameter (female: 19.8 ± 1.7% vs. male: 21.1 ± 2.0%; *p* < 0.001), left ventricle (LV) mass (female: 178.5 ± 59.7% vs. male: 204.3 ± 72.0%; *p* < 0.001), left ventricle end diastolic volume (LVEDV) (female: 101.9 ± 33.7 mL vs. male: 119.4 ± 44.8 mL; *p* < 0.001), and left ventricle end systolic volume (LVESV) (female: 39.5 ± 25.5 mL vs. male: 51.1 ± 36.6 mL; *p* < 0.001). On the contrary, female AS patients had higher functional measures of LVOT velocity time integral (VTI) (female: 59.6 ± 11.5 vs. male: 55.2 ± 14.3; *p* < 0.001), LVOT Vmax (female: 100.3 ± 22.9 cm/s vs. male: 91.6 ± 21.0 cm/s; *p* < 0.001), left atrial volume index (LAVI) (female: 40.0 ± 17.0 mL/m^2^ vs. male: 32.9 ± 13.6 mL/m^2^; *p* < 0.001), and left atrial (LA) area index (female: 13.2 ± 3.8 cm^2^/m^2^ vs. male: 11.2 ± 3.2 cm^2^/m^2^; *p* < 0.001). Significantly, female AS patients also had impaired diastology with a higher average E/e’ ratio (female: 17.8 ± 7.7 vs. male: 15.6 ± 9.1; *p* = 0.012) compared to male patients (Supplementary Table S1).

The mean follow-up duration was 4.1 ± 3.3 years and was similar for both male and female AS patients (*p* = 0.117). Fewer female AS patients underwent any aortic valve (AV) intervention (female: 21.2%, *n* = 84 vs. male: 27.8%, *n* = 85; *p* = 0.042) and surgical AV replacement (SAVR) (female: 11.3%, *n* = 45 vs. male: 18.6%, *n* = 57; *p* = 0.006) compared to male patients. Upon univariate analysis, a greater proportion of female AS patients encountered cardiovascular (CV) hospitalization during follow-up (female: 27.5%, *n* = 109 vs. male: 18.3%, *n* = 56; *p* = 0.005) compared to male patients, but there were no significant differences for the outcomes of subsequent heart failure (*p* = 0.612), stroke (*p* = 0.644), and all-cause mortality (*p* = 0.827).

**Figure 1:**
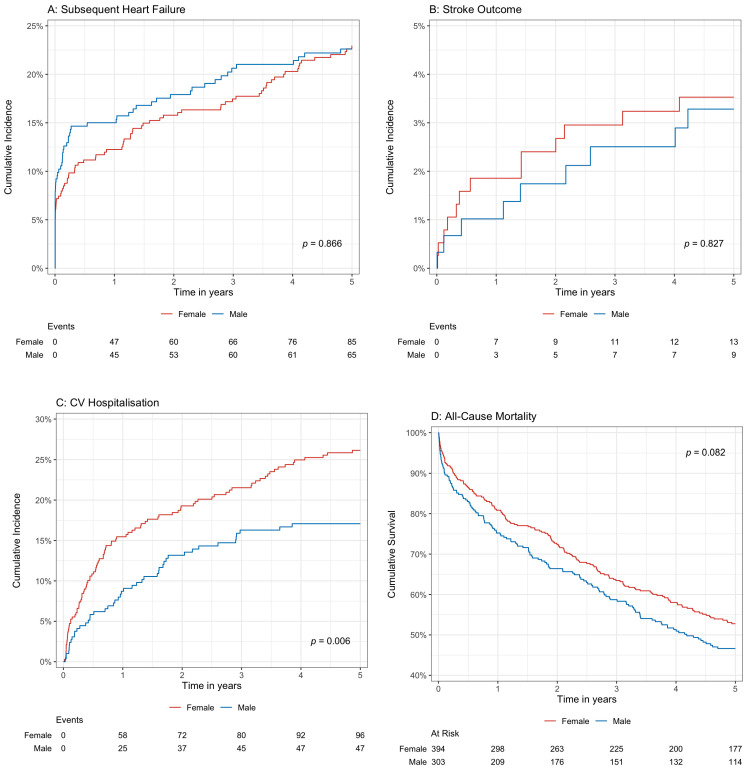
(**A**–**D**). Kaplan–Meier and cumulative incidence function estimates of outcomes comparing male and female aortic stenosis patients.

Survival analyses over a period of five years yielded similar results, with female AS patients having a higher incidence rate of CV hospitalization than male patients on cumulative incidence function (Figure 1C) (*p* = 0.006), but there were no significant differences for subsequent heart failure (*p* = 0.866), stroke outcome (*p* = 0.827), and all-cause mortality (*p* = 0.082) (Figure 1A,B,D, respectively). Subgroup analyses were then performed on moderate (*n* = 206) and severe (*n* = 109) AS patients (Table 2 and Supplementary Figures S1A–D and S2A–D).

In the moderate AS cohort, 53.4% (*n* = 110) were female with a mean age of 74.5 ± 13.3 years old. Fewer female patients with moderate AS had cardiovascular risk factors or diseases, such as current (*p* = 0.005) or previous (*p* < 0.001) smoking, CAD (*p* = 0.005), and previous AMI (*p* = 0.004) than male patients. There were no significant differences in terms of follow-up duration (*p* = 0.233), outcomes of AV intervention (*p* = 0.805), subsequent heart failure (*p* = 0.176), stroke (*p* >0.999), CV hospitalization (*p* = 0.799), and all-cause mortality (*p* = 0.760) upon univariate comparison and upon survival curve analyses (Supplementary Figure S1).

In the severe AS cohort, there was a similar proportion of female (50.5%, *n* = 55) and male patients (*n* = 54), with the mean age of female patients being 72.3 ± 12.8 years old. A greater proportion of female severe AS patients had atrial fibrillation (*p* = 0.032) than male patients, and there was no difference in medical therapy observed. However, fewer female patients with severe AS underwent SAVR (*p* = 0.020), and a greater proportion had CV hospitalization outcomes (*p* = 0.005). Cumulative incidence function estimates in severe AS patients (Supplementary Figure S2) again showed a higher incidence of CV hospitalization in female than male patients (*p* = 0.016), but there were no significant differences in subsequent heart failure (*p* = 0.660), stroke (*p* = 0.598), and all-cause mortality (*p* = 0.202).

In the multivariable competing risks (for all-cause mortality) regression model in the moderate AS cohort (Table 3), female sex was significantly associated with subsequent heart failure (aHR 2.01, 95% CI 1.06–3.83, *p* = 0.033), after adjusting for age, ethnicity, BMI, comorbidities, and LVEF.

In the severe AS cohort (Table 4), female sex remained an independent predictor for subsequent heart failure (aHR 2.89, 95% CI 1.01–8.29, *p* = 0.048) and CV hospitalization (aHR 20.0, 95% CI 1.19–335, *p* = 0.037), after adjusting for the same covariates as well as AV intervention.

## 4. Discussion

Findings in our cohort suggest that female Asian patients with AS undergoing both invasive and noninvasive AV intervention had significantly smaller BSA, fewer smokers, or ischemic heart disease; although, they had more anemia and chronic kidney disease. The anatomic dimensions of LVOT diameter, LV internal dimensions, end-systolic and end-diastolic volumes, and LV mass were smaller in female patients; but functional echocardiographic indices, such as aortic valve Vmax, VTI, and diastolic indices of LA area, volume, and E/e’ ratios, were higher in female patients. In terms of outcomes, female sex was a reliable predictor of cardiovascular hospitalizations in severe AS and an independent prognostic predictor of heart failure in both moderate and severe AS. There was no significant difference in stroke and all-cause mortality outcomes between sexes in Asian patients. Fewer female patients with severe AS also received AV intervention in our cohort, with a longer duration to AV intervention; although, AV intervention was shown to be protective of all-cause mortality in the female severe AS Asian cohort.

There is heterogeneity with regards to clinical outcomes between sex in AS, depending on the location, outcomes studied, and intervention performed reported in the literature [1,2]. Comparable to Western data, females at our center were older and tended to present later in the disease stage compared to males due to various factors, as described below [2]. Degeneration was the predominant etiology between sexes in our cohort. The sex-specific composition of aortic valvular degenerative stenosis showed a higher weightage of fibrosis in females compared to the greater proportion of calcification in males, with more leaflet-restricted mobility seen in females compared to males [3,7,8]. There were fewer coexisting cases of CAD and prior AMI amongst females, consistent with data from Western registries; although, the prevalence of coexisting cardiovascular risk factors of hypertension and hyperlipidemia were higher [2]. This could likely be accounted for by a shared driving force of multisystem atherosclerosis processes, resulting in valvular calcification, inflammatory processes, and fibro-fatty buildup in both AS and IHD [1,4]. More severe grades of anemia were present in females with AS, explained by poorer iron absorption and hepcidin elevation associated with inflammation in atherosclerosis, and increased incidences of chronic kidney disease were seen in females with AS due to a shared driving mechanism between kidney dysfunction and accelerated atherosclerosis in AS [9].

Female characteristics in our Asian cohort have biological physiques of smaller body surface areas, rendering smaller left ventricular outflow tract measurements, left ventricular internal dimensions, left ventricular volumes in both systole and diastole, and a left ventricular mass similar to our Western counterparts [3]. Stroke volume is generally smaller and the flow rate typically lower in females compared to males, manifesting with higher prevalence of paradoxical low flow low gradient AS [3]. With regards to physiological adaptations, there have been reports of a preferentially smaller chamber size and inward concentric remodeling of the LV cavity in women compared to the more eccentric remodeling seen in men that result in larger chamber sizes [3]. Increased diastolic dysfunction that has been reported in the literature was also observed in our cohort due to restricted cavity size, increased stiffness, decreased compliance, and subsequent ventriculo-arterial impedance [1]. This leads to increased wall stress, elevated filling pressures, and consequently, higher left atrial volumes and pulmonary pressures [1]. Similar to studies that have suggested a higher occurrence of low ejection fraction in males and suggestions of higher LVEF in females in the Western cohort, there have been proposals for a gender-based LVEF cutoff [10]. The above ventricular changes are favorable for clinical compensation seen in women that could account for the increased symptomology, later clinical course at presentation of the disease, and increased presentation with heart failure [11].

The abovementioned sex-specific differences in cardiac morphology and physiological adaptations are multifactorial and helmed by genetic, hormonal, and cellular mechanisms [3]. The hormones of testosterone and 17β-estradiol-activating estrogen receptors to upregulate profibrotic and inflammatory gene expression of collagens I and III result in fibrosis and cardiac apoptosis processes in the left ventricular remodeling seen in males [3]. In females, matrix-metalloproteinase 2 gene expression, preferential myocardial transcriptional activation of collagen I, polymorphism in the estrogen receptor, and the functional polymorphism of the renalase (RNL) gene to reduce catecholamines and transforming growth factor-β signaling pathways results in the adaptive ventricular changes seen in females [3,12,13,14,15,16]. The different activation pathways in males and females lead to the distinct gender profiles of clinical phenotypes, remodeling manifestations, and hemodynamic responses in AS that could subsequently affect treatment timing and uptake [2].

With regards to follow-up and outcome, females were followed-up for a longer period of time compared to males, likely due to increased life expectancy established in epidemiological studies and the literature [17]. Subsequent heart failure episodes were observed in all grades of AS in the female cohort compared to the male cohort to a more severe degree in the severe AS group compared to the moderate AS group in view of the culmination of hemodynamic stress in the more advanced stages of AS. Cardiovascular hospitalizations were also significantly higher in the severe AS cohort compared to the moderate AS cohort, in view of physiological compensation until the later stages of the disease. In spite of increased cardiovascular hospitalizations seen in females, there were no significant differences in terms of all-cause mortality, which is comparable to the literature on the Western population. However, fewer females received AV intervention with a longer duration experienced until AV intervention. This might be attributed to the atypicality of presenting symptoms in the presence of higher incidences of microvascular dysfunction [16,18,19,20,21,22,23]. The physiological variances of smaller aortic root, LV cavity, smaller stroke volume index, and lower flow rate leads to the under-diagnosis and under-estimation of valvular severity [3]. On top of delayed presentation and under-recognition, the perception of higher intra-operative risks that have been incorporated into formal risk scores of EuroScore and STS risk scores is factored into the increased tendency for females to turn down valvular intervention. This is similar to our Western cohort where a significantly lower number of patients underwent aortic valvular intervention, especially surgical intervention, with a longer duration experienced due to the receipt of aortic valve intervention [20,21,22].

Similar to Western counterparts, female sex was an independent risk factor for heart failure due to the baseline elevated filling pressures, diastolic dysfunction, and microvascular epicardial dysfunction [23]. Asian females had higher levels of hospitalizations and worse outcomes as a result of concomitant heart, vascular, and pulmonary disease in heart failure [17,23].

In the severe AS population, AV intervention reduced all-cause mortality, due to the brisk nature of negative remodeling and the regression of hypertrophy reported in females after AV intervention [3,22,24,25,26]. Prognosis was largely determined by the offloading of left ventricular outflow tract obstruction and pressure overload reversal with its cascade hemodynamic effects in this cohort [24]. Post-AV intervention, there have been reports of worse short-term outcomes of bleeding but improved long-term survival of stroke and mortality in females [3].

WIN-TAVI was a dedicated female registry to spearhead the study of TAVR in the intermediate- to high-risk group to show Valve Academic Research Consortium (VARC)-2 composite efficacy endpoint with low rates of stroke and mortality at 1 year [22]; while RHEIA, which compared TAVR with SAPIEN 3 or SAPIEN 3 ULTRA to SAVR in females with all-comer AS, showed that TAVR was superior to SAVR for the primary composite endpoint of death, stroke, and rehospitalization at 1 year [27]. This could pave the way for new potential guidelines to mitigate observed disparities and narrow sex-specific AS management gaps in the literature.

In the absence of conservative or pharmacological measures to delay the progression of AS, novel therapeutics, such as sodium–glucose cotransporter-2 inhibitors (SGLT2i) have emerged in the context of AS with other indications, such as diabetes, reduced left ventricular ejection fraction, and in those undergoing TAVR [28,29].

With a better understanding of the pathophysiology behind sex-specific drivers of aortic stenosis, streamlined echocardiographic diagnostic features, and clinical outcome differences, there should be an adoption of a heightened index of suspicion with a lower threshold for the diagnosis of symptomatic AS and timely referral for prompt intervention where it is indicated that can alter mortality in Asian females [7]. Further work needs to be carried out for the consideration of sex-specific cutoffs for severity grading and AV intervention referral in this cohort in the future.

To the best of our knowledge, a strength of this study was that it described one of the longest longitudinal outcomes between genders in a large Asian cohort over 10 years. A limitation of this study was that it is a retrospective observational study with inherent risks of selection bias confounding variables. Also, we could only demonstrate association and not causation.

## 5. Conclusions

In Asian patients with AS, female patients were older and more symptomatic at the time of presentation. Female sex was an independent prognostic predictor of heart failure in both moderate and severe AS undergoing both invasive and noninvasive AV intervention and associated with cardiovascular hospitalizations in severe AS. There were more cardiovascular hospitalizations, longer duration to AV intervention for females with moderate-to-severe AS, and fewer surgical aortic valve intervention uptake in females with severe AS in our cohort. AV intervention is shown to be protective of all-cause mortality in our female severe AS cohort. However, there was no difference in stroke and all-cause mortality outcomes between male and female Asian patients with moderate-to-severe AS. Further studies are required to determine the prognostic and therapeutic implications of sex in Asian patients with AS [30].

## Figures and Tables

**Table 1:** Baseline characteristics of all aortic stenosis patients stratified by sex.

Variables	*n*	Overall *n* = 703	Male *n* = 306	Female *n* = 397	*p*-Value
Baseline Demographics					
Age, mean (SD)	703	74.4 (12.3)	73.5 (12.7)	75.1 (12.0)	0.084
Ethnicity, *n* (%)					0.451
Chinese		437 (62.2)	185 (60.5)	252 (63.5)	
Malay		112 (15.9)	54 (17.6)	58 (14.6)	
Indian		61 (8.7)	23 (7.5)	38 (9.6)	
Others		93 (13.2)	44 (14.4)	49 (12.3)	
BSA (m^2^), mean (SD)		1.6 (0.2)	1.7 (0.2)	1.6 (0.2)	<0.001
BMI (kg/m^2^), mean (SD)		24.8 (5.4)	24.4 (5.1)	25.2 (5.6)	0.051
Clinical Findings					
SOB (NYHA Class), *n* (%)	345				0.043
Class 1		136 (39.5)	65 (44.2)	71 (35.9)	
Class 2		96 (27.9)	42 (28.6)	54 (27.3)	
Class 3		70 (20.3)	30 (20.4)	40 (20.2)	
Class 4		43 (12.5)	10 (6.8)	33 (16.7)	
Comorbidities, *n* (%)	703				
Current smoker		86 (12.2)	61 (19.9)	25 (6.3)	<0.001
Previous smoker		133 (18.9)	98 (32.0)	35 (8.8)	<0.001
Hypertension		535 (76.1)	224 (73.2)	311 (78.3)	0.113
Hyperlipidemia		439 (62.4)	182 (59.5)	257 (64.7)	0.153
Diabetes mellitus		280 (39.8)	119 (38.9)	161 (40.6)	0.655
Coronary artery disease		306 (43.5)	153 (50.0)	153 (38.5)	0.002
Previous AMI		160 (22.8)	83 (27.1)	77 (19.4)	0.015
Heart failure		87 (12.4)	37 (12.1)	50 (12.6)	0.841
Atrial fibrillation		132 (18.8)	50 (16.3)	82 (20.7)	0.146
Previous stroke/TIA		120 (17.1)	55 (18.0)	65 (16.4)	0.576
COPD		29 (4.1)	19 (6.2)	10 (2.5)	0.015
Malignancy		78 (11.1)	27 (8.8)	51 (12.8)	0.092
Anemia		387 (55.0)	163 (53.3)	224 (56.4)	0.404
CKD	670	264 (39.4)	103 (34.7)	161 (43.2)	0.026
ESKD		61 (9.1)	22 (7.4)	39 (10.5)	0.173
Hemoglobin (g/dL), mean (SD)	660	11.8 (2.2)	12.4 (2.3)	11.4 (2.0)	<0.001
Platelet (×10^9^/L), mean (SD)	659	239 (90)	222 (83)	252 (94)	<0.001
Creatinine (μmol/L), mean (SD)	669	131 (150)	139 (155)	123 (145)	0.222
GDMT, *n* (%)	703				
Aspirin		313 (44.5)	151 (49.3)	162 (40.8)	0.024
OAC		83 (11.8)	32 (10.5)	51 (12.8)	0.330
Statins		413 (58.7)	178 (58.2)	235 (59.2)	0.785
ACEi or ARB		163 (23.2)	66 (21.6)	97 (24.4)	0.372
BB		272 (38.7)	129 (42.2)	143 (36.0)	0.098
Echocardiogram Findings					
AS severity, *n* (%)	703				0.126
Mild		388 (55.2)	156 (51.0)	232 (58.4)	
Moderate		206 (65.4)	96 (64.0)	110 (66.7)	
Severe		109 (34.6)	54 (36.0)	55 (33.3)	
AS etiology, *n* (%)	613				
Calcific/degenerative		555 (90.5)	249 (90.9)	306 (90.3)	0.797
Bicuspid		58 (9.5)	21 (7.7)	37 (10.9)	0.172
Rheumatic		24 (3.9)	13 (4.7)	11 (3.2)	0.341
Others		4 (0.7)	0 (0.0)	4 (1.2)	0.132
AS flow state, *n* (%)	206				0.229
Paradoxical LFLG		24 (11.7)	10 (11.6)	14 (11.7)	
Classical LFLG		30 (14.6)	14 (16.3)	16 (13.3)	
NFLG		56 (27.2)	17 (19.8)	39 (32.5)	
HFHG		96 (46.6)	45 (52.3)	51 (42.5)	
AVA (cm^2^), mean (SD) AVA index (cm^2^/m^2^), mean (SD)	693	1.14 (0.41) 0.71 (0.27)	1.14 (0.38) 0.68 (0.24)	1.14 (0.43) 0.74 (0.28)	0.849 0.009
MPG (mmHg), mean (SD)		24.0 (17.1)	25.2 (17.8)	23.1 (16.5)	0.106
PPG (mmHg), mean (SD)		40.6 (25.8)	41.6 (25.7)	39.8 (26.0)	0.364
Vmax (m/sec), mean (SD)		2.9 (0.9)	3.0 (0.9)	3.0 (0.9)	0.481
Stroke volume (mL), mean (SD) Stroke volume index (mL/m^2^), mean (SD)		65.0 (20.9) 40.3 (12.5)	68.2 (22.6) 40.5 (13.1)	62.5 (19.1) 40.2 (12.0)	<0.001 0.780
DI, mean (SD)		0.3 (0.1)	0.3 (0.1)	0.4 (0.2)	<0.001
LVOT diameter (mm), mean (SD)		20.4 (1.9)	21.1 (2.0)	19.8 (1.7)	<0.001
LVOT VTI (mm), mean (SD)		21.3 (7.0)	20.1 (5.5)	22.3 (7.8)	<0.001
LVOT Vmax (cm/sec), mean (SD)		96.5 (22.5)	91.6 (21.0)	100.3 (22.9)	<0.001
LVEF (%), mean (SD)	556	57.8 (12.9)	55.2 (14.3)	59.6 (11.5)	<0.001
RWMA, *n* (%)	433	126 (29.1)	78 (40.2)	48 (20.1)	<0.001
LV mass (g), mean (SD) LV mass index (g/m^2^), mean (SD)	693	189.9 (66.6) 117.4 (39.5)	204.3 (72.0) 120.6 (40.2)	178.5 (59.7) 114.8 (38.8)	<0.001 0.057
LVIDd (mm), mean (SD) LVIDs (mm), mean (SD)		47.7 (7.2) 31.7 (8.3)	49.5 (7.7) 33.5 (9.1)	46.3 (6.5) 30.3 (7.3)	<0.001 <0.001
IVSs (mm), mean (SD) IVSd (mm), mean (SD)		10.8 (2.7) 14.7 (3.2)	10.9 (2.8) 15.0 (3.2)	10.7 (2.7) 14.5 (3.1)	0.537 0.054
LVPWd (mm), mean (SD) LVPWs (mm), mean (SD)		10.5 (2.0) 14.8 (2.7)	10.6 (2.1) 15.0 (2.8)	10.4 (1.9) 14.6 (2.6)	0.387 0.091
LVEDV (mL), mean (SD) LVEDV index (mL/m^2^), mean (SD)		109.6 (39.9) 68.0 (24.4)	119.4 (44.8) 69.9 (26.1)	101.9 (33.7) 66.5 (22.8)	<0.001 0.070
LVESV (mL), mean (SD) LVESV index (mL/m^2^), mean (SD)		44.6 (31.4) 27.7 (19.1)	51.1 (36.6) 30.4 (21.7)	39.5 (25.5) 25.6 (16.5)	<0.001 <0.001
LA volume (mL), mean (SD) LA volume index (mL/m^2^), mean (SD)	418	59.4 (24.5) 36.8 (16.0)	56.2 (23.2) 32.9 (13.6)	61.9 (25.2) 40.0 (17.0)	0.019 <0.001
LA area (cm^2^), mean (SD) LA area index (cm^2^/m^2^), mean (SD)		19.9 (5.5) 12.3 (3.7)	19.2 (5.5) 11.2 (3.2)	20.4 (5.5) 13.2 (3.8)	0.026 <0.001
EA, mean (SD)	584	1.1 (1.8)	1.0 (0.7)	1.1 (2.4)	0.630
Septal E/e’, mean (SD)	629	20.4 (12.5)	18.8 (11.1)	21.7 (13.3)	0.003
Lateral E/e’, mean (SD)	390	14.8 (7.8)	13.7 (8.4)	15.6 (7.2)	0.016
Average E/e’, mean (SD)	387	16.9 (8.4)	15.6 (9.1)	17.8 (7.7)	0.012
PASP (mmHg), mean (SD)	617	38.9 (15.0)	37.8 (14.9)	39.8 (15.0)	0.101
Outcomes					
Follow-up duration (years), mean (SD)	697	4.1 (3.3)	3.9 (3.4)	4.3 (3.2)	0.117
AV intervention, n (%)	703	169 (24.0)	85 (27.8)	84 (21.2)	0.042
SAVR		102 (14.5)	57 (18.6)	45 (11.3)	0.006
TAVR		75 (10.7)	34 (11.1)	41 (10.3)	0.739
Duration to AV intervention (years), mean (SD)	129	3.0 (2.3)	2.4 (2.2)	3.6 (2.4)	0.004
Subsequent HF, *n* (%)	703	172 (24.5)	72 (23.5)	100 (25.2)	0.612
Stroke outcome, *n* (%)		28 (4.0)	11 (3.6)	17 (4.3)	0.644
CV hospitalization, *n* (%)		165 (23.5)	56 (18.3)	109 (27.5)	0.005
All-cause mortality, *n* (%)		431 (61.3)	189 (61.8)	242 (61.0)	0.827

Abbreviations:,ACEi—angiotensin-converting enzyme inhibitors, AMI—acute myocardial infarction, ARB—angiotensin receptor blockers, AS—aortic stenosis, AV—aortic valve, AVA—aortic valve area, BMI—body mass index, BSA—body surface area, CKD—chronic kidney disease, COPD—chronic obstructive pulmonary disease, CV—cardiovascular, GDMT—goal directed medical therapy, HFHG—high flow high gradient, IVS—interventricular septum, LA—left atrium, LFLG—low flow low gradient, LV—left ventricular, LVID—left ventricular internal diameter, LVEDV—left ventricular end diastolic volume, LVESV—left ventricular end systolic volume, LVOT—left ventricular outflow tract, LVPW—left ventricular posterior wall diameter, MPG—mean pressure gradient, NFLG—normal flow low gradient, NYHA—New York Heart Association classification, OAC—oral anticoagulation, PASP—pulmonary artery systolic pressure, PPG—peak pressure gradient, RWMA—regional wall motion abnormality, SAVR—surgical aortic valve replacement, TAVR—transcatheter aortic valve replacement, TIA—transient ischemic attack, VTI—velocity time integral.

**Table 2:** Clinical characteristics of aortic stenosis patients stratified by sex and severity (moderate and severe).

Variables	Moderate AS				Severe AS			
	*n*	Male *n* = 96	Female *n* = 110	*p*-Value	*n*	Male *n* = 54	Female *n* = 55	*p*-Value
Baseline Demographics								
Age, mean (SD)	206	71.4 (12.9)	74.5 (13.3)	0.092	109	68.9 (12.8)	72.3 (12.8)	0.173
Chinese		60 (62.5)	66 (60.0)			29 (53.7)	42 (76.4)	
Malay		15 (15.6)	16 (14.5)			10 (18.5)	4 (7.3)	
Indian		4 (4.2)	8 (7.3)			5 (9.3)	5 (9.1)	
Others		17 (17.7)	20 (18.2)			10 (18.5)	4 (7.3)	
BSA (m^2^), mean (SD)		1.7 (0.2)	1.6 (0.2)	<0.001		1.7 (0.2)	1.5 (0.2)	<0.001
BMI (kg/m^2^), mean (SD)		25.3 (6.4)	26.9 (6.4)	0.072		24.0 (3.9)	23.9 (5.0)	0.928
Clinical Findings								
Comorbidities, *n* (%)	206				109			
Current smoker		17 (17.7)	6 (5.5)	0.005		11 (20.4)	4 (7.3)	0.047
Previous smoker		25 (26.0)	8 (7.3)	<0.001		13 (24.1)	4 (7.3)	0.016
Hypertension		68 (70.8)	88 (80.0)	0.126		36 (66.7)	32 (58.2)	0.361
Hyperlipidemia		55 (57.3)	66 (60.0)	0.694		28 (51.9)	38 (69.1)	0.066
Diabetes mellitus		39 (40.6)	41 (37.3)	0.622		15 (27.8)	18 (32.7)	0.574
Coronary artery disease		46 (47.9)	32 (29.1)	0.005		21 (38.9)	22 (40.0)	0.906
Previous AMI		24 (25.0)	11 (10.0)	0.004		10 (18.5)	8 (14.5)	0.576
Heart failure		19 (19.8)	13 (11.8)	0.115		7 (13.0)	10 (18.2)	0.453
Atrial fibrillation		19 (19.8)	12 (10.9)	0.075		6 (11.1)	15 (27.3)	0.032
Previous stroke/TIA		14 (14.6)	13 (11.8)	0.557		3 (5.6)	6 (10.9)	0.489
COPD		7 (7.3)	4 (3.6)	0.244		3 (5.6)	1 (1.8)	0.363
Malignancy		9 (9.4)	11 (10.0)	0.880		4 (7.4)	5 (9.1)	>0.999
Anemia		56 (58.3)	54 (49.1)	0.185		27 (50.0)	25 (45.5)	0.635
CKD		35 (38.0)	39 (39.0)	0.892		15 (28.8)	21 (40.4)	0.216
ESKD	192	8 (8.7)	11 (11.0)	0.593	104	1 (1.9)	4 (7.7)	0.363
Hemoglobin (g/dL), mean (SD)	191	12.4 (2.1)	11.4 (2.1)	0.002	105	12.8 (2.3)	11.7 (1.4)	0.004
Platelet (×10^9^/L), mean (SD)		219.1 (68.5)	257.3 (99.0)	0.002		212.3 (85.5)	241.6 (84.0)	0.089
Creatinine (μmol/L), mean (SD)		157.7 (169.7)	114.3 (131.1)	0.047		106.2 (128.3)	109.5 (99.2)	0.883
GDMT, *n* (%)	206				109			
Aspirin		43 (44.8)	47 (42.7)	0.766		22 (40.7)	23 (41.8)	0.909
OAC		17 (17.7)	9 (8.2)	0.040		5 (9.3)	10 (18.2)	0.176
Statins		58 (60.4)	70 (63.6)	0.635		27 (50.0)	30 (54.5)	0.635
ACEi or ARB		20 (20.8)	23 (20.9)	0.989		12 (22.2)	10 (18.2)	0.599
BB		39 (40.6)	40 (36.4)	0.530		24 (44.4)	18 (32.7)	0.209
Echocardiogram Findings								
AS etiology, *n* (%)	177				91			
Calcific/degenerative		70 (86.4)	89 (92.7)	0.168		44 (93.6)	38 (86.4)	0.306
Bicuspid		9 (11.1)	8 (8.3)	0.532		2 (4.3)	7 (15.9)	0.084
Rheumatic		4 (4.9)	2 (2.1)	0.414		1 (2.1)	2 (4.5)	0.608
AVA (cm^2^), mean (SD) AVA index (cm^2^/m^2^), mean (SD)	206	1.1 (0.3) 0.6 (0.2)	1.0 (0.3) 0.6 (0.2)	0.378 0.476	109	0.7 (0.2) 0.4 (0.1)	0.7 (0.2) 0.4 (0.1)	0.097 0.811
MPG (mmHg), mean (SD)		28.4 (7.9)	26.9 (5.6)	0.107		55.6 (14.7)	56.2 (14.5)	0.831
PPG (mmHg), mean (SD)		47.9 (16.3)	46.7 (16.8)	0.612		80.9 (22.3)	83.8 (26.8)	0.533
Vmax (m/sec), mean (SD)		337.1 (53.1)	327.9 (62.8)	0.279		439.7 (69.5)	449.4 (80.3)	0.515
Stroke volume (mL), mean (SD) Stroke volume index (mL/m^2^), mean (SD)		69.7 (22.5) 41.0 (13.1)	65.8 (20.4) 41.1 (12.1)	0.195 0.986		68.6 (26.3) 40.2 (14.4)	63.7 (19.9) 41.6 (12.1)	0.281 0.586
DI, mean (SD)		0.3 (0.1)	0.3 (0.1)	0.015		0.2 (0.1)	0.2 (0.1)	0.179
LVOT diameter (mm), mean (SD)		21.7 (2.1)	20.0 (1.6)	<0.001		21.4 (2.2)	20.3 (1.9)	0.008
LVOT VTI (mm), mean (SD)		20.9 (5.7)	23.2 (6.7)	0.009		20.7 (5.9)	23.3 (6.5)	0.032
LVOT Vmax (m/sec), mean (SD)		94.5 (20.6)	103.4 (26.2)	0.008		88.2 (21.0)	97.6 (25.0)	0.036
LVEF (%), mean (SD)		54.9 (15.4)	60.6 (12.3)	0.004		52.1 (14.4)	56.1 (12.9)	0.137
RWMA, *n* (%)	114	20 (33.9)	4 (7.3)	<0.001	20	4 (36.4)	3 (33.3)	>0.999
LV mass (g), mean (SD) LV mass index (g/m^2^), mean (SD)	206	216.4 (68.5) 127.0 (37.4)	190.1 (66.0) 118.8 (40.8)	0.006 0.141	109	244.1 (80.4) 143.7 (47.3)	214.8 (60.3) 140.4 (39.0)	0.035 0.697
LVIDd (mm), mean (SD) LVIDs (mm), mean (SD)		50.4 (8.2) 34.4 (10.0)	46.9 (7.0) 30.2 (7.9)	0.001 <0.001		49.9 (7.9) 34.2 (8.8)	47.5 (6.3) 31.9 (7.3)	0.093 0.143
IVSs (mm), mean (SD) IVSd (mm), mean (SD)		10.9 (3.1) 15.1 (3.1)	10.4 (2.9) 15.0 (3.1)	0.297 0.817		10.8 (3.3) 16.4 (3.1)	10.4 (1.8) 15.7 (2.9)	0.375 0.227
LVPWd (mm), mean (SD) LVPWs (mm), mean (SD)		10.8 (1.7) 15.1 (2.6)	10.8 (1.6) 14.8 (2.4)	0.910 0.454		12.0 (2.5) 16.4 (3.0)	11.7 (2.3) 16.1 (2.9)	0.527 0.604
LVEDV (mL), mean (SD) LVEDV index (mL/m^2^), mean (SD)		125.6 (47.9) 72.1 (27.3)	105.2 (37.5) 66.8 (24.5)	<0.001 0.143		121.6 (44.9) 69.8 (24.0)	107.6 (35.0) 71.6 (25.0)	0.075 0.708
LVESV (mL), mean (SD) LVESV index (mL/m^2^), mean (SD)		55.2 (42.1) 32.5 (24.2)	39.5 (29.7) 24.8 (18.6)	0.002 0.012		52.9 (34.5) 31.1 (20.2)	43.9 (26.7) 28.6 (16.8)	0.130 0.492
LA volume (mL), mean (SD) LA volume index (mL/m^2^), mean (SD)	112	61.8 (27.5) 35.2 (15.6)	63.0 (20.4) 39.4 (12.9)	0.789 0.125	19	56.3 (24.6) 31.1 (12.6)	52.2 (20.2) 31.3 (11.2)	0.702 0.973
LA area (cm^2^), mean (SD) LA area index (cm^2^/m^2^), mean (SD)		20.4 (6.3) 11.7 (3.5)	20.7 (4.4) 13.0 (2.9)	0.785 0.034		19.4 (5.4) 10.7 (2.9)	18.4 (4.8) 11.1 (2.6)	0.686 0.790
EA, mean (SD)	177	1.0 (0.6)	0.9 (0.5)	0.209	93	0.9 (0.4)	1.1 (0.5)	0.044
Septal E/e’, mean (SD)	187	20.0 (12.2)	21.2 (12.5)	0.512	100	22.1 (15.0)	24.5 (16.4)	0.461
Lateral E/e’, mean (SD)	106	15.8 (10.3)	14.8 (6.8)	0.578	17	10.9 (3.0)	20.0 (7.1)	0.002
Average E/e’, mean (SD)	106	18.2 (11.8)	17.2 (7.3)	0.595	17	13.8 (6.0)	19.4 (6.3)	0.084
PASP (mmHg), mean (SD)	188	38.4 (16.2)	36.7 (13.9)	0.449	99	38.0 (16.4)	42.4 (16.6)	0.183
Outcomes								
Follow-up duration (years), mean (SD)	206	4.0 (3.3)	4.5 (3.4)	0.233	109	4.4 (3.7)	4.1 (3.6)	0.687
AV intervention, *n* (%)		25 (26.0)	27 (24.5)	0.805		25 (46.3)	17 (30.9)	0.099
SAVR		15 (15.6)	17 (15.5)	0.973		23 (42.6)	12 (21.8)	0.020
TAVR		10 (10.4)	10 (9.1)	0.749		6 (11.1)	6 (10.9)	0.973
Duration to AV intervention (years), mean (SD)	43	2.1 (1.9)	3.2 (2.3)	0.108	19	1.9 (2.3)	3.0 (2.2)	0.297
Subsequent HF, *n* (%)	206	24 (25.0)	37 (33.6)	0.176	109	19 (35.2)	16 (29.1)	0.496
Stroke outcome, *n* (%)		3 (3.1)	3 (2.7)	>0.999		3 (5.6)	4 (7.3)	>0.999
CV hospitalization, *n* (%)		17 (17.7)	21 (19.1)	0.799		5 (9.3)	17 (30.9)	0.005
All-cause mortality, *n* (%)		57 (59.4)	63 (57.3)	0.760		24 (44.4)	32 (58.2)	0.151

**Table 3:** Multivariable regression models for outcomes in moderate aortic stenosis patients.

Variables	Subsequent Heart Failure ^1^		CV Hospitalisation ^1^		All-Cause Mortality ^2^	
	aHR (95% CI) ^3^	*p*-Value	aHR (95% CI) ^3^	*p*-Value	aHR (95% CI) ^3^	*p*-Value
Age (per year)	0.98 (0.96 to 1.00)	0.065	1.02 (0.98 to 1.06)	0.278	1.02 (0.99 to 1.04)	0.134
Female sex	2.01 (1.06 to 3.83)	0.033	1.69 (0.74 to 3.87)	0.216	0.94 (0.58 to 1.51)	0.795
Ethnicity		1.000		1.000		0.843
Chinese	Reference		Reference		Reference	
Malay	1.21 (0.59 to 2.49)		1.40 (0.57 to 3.40)		0.86 (0.47 to 1.57)	
Indian	N/A		N/A		0.75 (0.23 to 2.47)	
Others	0.60 (0.22 to 1.64)		2.17 (0.75 to 6.28)		1.21 (0.57 to 2.58)	
BMI (per kg/m^2^)	1.01 (0.97 to 1.05)	0.569	0.99 (0.93 to 1.05)	0.772	0.98 (0.94 to 1.03)	0.436
CAD	2.39 (1.25 to 4.58)	0.009	1.22 (0.56 to 2.66)	0.612	1.14 (0.71 to 1.83)	0.578
Previous stroke or TIA	0.87 (0.40 to 1.88)	0.720	2.78 (1.12 to 6.88)	0.027	1.54 (0.87 to 2.75)	0.154
CKD	0.76 (0.38 to 1.54)	0.451	2.16 (1.04 to 4.48)	0.038	2.76 (1.72 to 4.44)	<0.001
Anemia	0.82 (0.43 to 1.58)	0.555	0.94 (0.43 to 2.03)	0.871	2.91 (1.71 to 4.96)	<0.001
LVEF (per 5%)	0.87 (0.78 to 0.96)	0.006	0.94 (0.82 to 1.07)	0.334	0.94 (0.87 to 1.01)	0.115

^1^ Fine and Gray competing risks model (for mortality); ^2^ Cox proportional hazards model; ^3^ aHR = adjusted hazard ratio, CI = confidence interval, N/A = no events for subgroup due to small sample size.

**Table 4:** Multivariable regression models for outcomes in severe aortic stenosis patients.

Variables	AV Intervention ^1^		Subsequent Heart Failure ^1^		CV Hospitalisation ^1^		All-Cause Mortality ^2^	
	aHR (95% CI) ^3^	*p*-Value	aHR (95% CI) ^3^	*p*-Value	aHR (95% CI) ^3^	*p*-Value	aHR (95% CI) ^3^	*p*-Value
Age (per year)	1.00 (0.96 to 1.04)	0.842	1.00 (0.95 to 1.04)	0.892	1.03 (0.98 to 1.08)	0.232	1.04 (1.00 to 1.09)	0.041
Female sex	0.29 (0.08 to 1.14)	0.077	2.89 (1.01 to 8.29)	0.048	20.0 (1.19 to 335)	0.037	1.18 (0.59 to 2.37)	0.644
Ethnicity		0.926		<0.001		0.008		0.527
Chinese	Reference		Reference		Reference		Reference	
Malay	0.68 (0.14 to 3.24)		3.63 (1.02 to 12.9)		21.3 (3.71 to 122)		0.53 (0.18 to 1.55)	
Indian	0.85 (0.10 to 7.02)		1.16 (0.25 to 5.48)		0.71 (0.01 to 40.9)		1.62 (0.41 to 6.50)	
Others	1.43 (0.21 to 9.77)		14.6 (3.95 to 53.9)		1.40 (0.18 to 10.7)		0.90 (0.29 to 2.83)	
BMI (per kg/m^2^)	1.01 (0.91 to 1.13)	0.828	1.05 (0.95 to 1.15)	0.339	0.97 (0.84 to 1.11)	0.638	0.93 (0.85 to 1.01)	0.060
CAD	1.85 (0.55 to 6.16)	0.318	0.79 (0.34 to 1.83)	0.581	1.52 (0.31 to 7.43)	0.608	0.74 (0.38 to 1.47)	0.392
Previous stroke or TIA	1.27 (0.10 to 15.8)	0.852	0.46 (0.12 to 1.82)	0.269	12.3 (1.86 to 81.4)	0.009	2.03 (0.63 to 6.53)	0.257
CKD	1.14 (0.30 to 4.34)	0.845	1.27 (0.49 to 3.30)	0.623	5.72 (0.91 to 35.9)	0.063	2.49 (1.17 to 5.31)	0.017
Anemia	0.70 (0.26 to 1.90)	0.484	2.72 (1.11 to 6.66)	0.028	0.97 (0.17 to 5.43)	0.975	0.72 (0.35 to 1.50)	0.383
LVEF (per 5%)	1.07 (0.85 to 1.35)	0.556	1.02 (0.86 to 1.20)	0.837	0.89 (0.71 to 1.13)	0.348	0.87 (0.76 to 1.00)	0.045
AV intervention	N/A	N/A	0.28 (0.05 to 1.71)	0.168	2.41 (0.27 to 21.5)	0.432	0.18 (0.07 to 0.48)	<0.001

^1^ Fine and Gray competing risks model (for mortality); ^2^ Cox proportional hazards model; ^3^ aHR = adjusted hazard ratio, CI = confidence interval, N/A = covariate not included in multivariable model.

## Data Availability

The original contributions presented in this study are included in the article/Supplementary Materials.

## Supplementary Materials

The following supporting information can be downloaded at: https://www.mdpi.com/article/10.3390/jcdd12010032/s1. Table S1. Echocardiographic parameters of all aortic stenosis patients stratified by sex. Table S2. Echocardiographic parameters of aortic stenosis patients stratified by sex and severity (moderate and severe). Figure S1A–D. Kaplan-Meier and Cumulative Incidence Function estimates of outcomes comparing male and female moderate aortic stenosis patients. Figure S2A–D. Kaplan-Meier and Cumulative Incidence Function estimates of outcomes comparing male and female severe aortic stenosis patients.
