# Supplementary material for: Longitudinal Outcomes of Patients with Aortic Stenosis Stratified by Sex: An Asian Perspective
Source: J Cardiovasc Dev Dis. 2025 Jan 19;12(1):32. doi: 10.3390/jcdd12010032 (PMC11766203; doi:10.3390/jcdd12010032)
Supplement: Supplementary file 1 [file jcdd-12-00032-s001.zip › jcdd-3381845-supplementary.pdf]

Table S1. Echocardiographic parameters of all aortic stenosis patients stratified by sex.

Variables	*n*	Overall<br>*n* = 703	Male<br>*n* = 306	Female<br>*n* = 397	*p*-value
AVA (cm^2), mean (SD)	693	1.14 (0.41)	1.14 (0.38)	1.14 (0.43)	0.849
AVA index (cm^2/m^2), mean (SD)		0.71 (0.27)	0.68 (0.24)	0.74 (0.28)	**0.009**
MPG (mmHg), mean (SD)		24.0 (17.1)	25.2 (17.8)	23.1 (16.5)	0.106
PPG (mmHg), mean (SD)		40.6 (25.8)	41.6 (25.7)	39.8 (26.0)	0.364
Vmax (m/sec), mean (SD)		293.3 (99.9)	299.4 (95.1)	288.7 (103.2)	0.166
Stroke volume (ml), mean (SD)		65.0 (20.9)	68.2 (22.6)	62.5 (19.1)	**<0.001**
Stroke volume index (ml/m^2), mean (SD)		40.3 (12.5)	40.5 (13.1)	40.2 (12.0)	0.780
DI, mean (SD)		0.3 (0.1)	0.3 (0.1)	0.4 (0.2)	**<0.001**
LVOT diameter (mm), mean (SD)		20.4 (1.9)	21.1 (2.0)	19.8 (1.7)	**<0.001**
LVOT VTI (mm), mean (SD)		21.3 (7.0)	20.1 (5.5)	22.3 (7.8)	**<0.001**
LVOT Vmax (m/sec), mean (SD)		96.5 (22.5)	91.6 (21.0)	100.3 (22.9)	**<0.001**
LVEF (%), mean (SD)	556	57.8 (12.9)	55.2 (14.3)	59.6 (11.5)	**<0.001**
RWMA, *n* (%)	433	126 (29.1)	78 (40.2)	48 (20.1)	**<0.001**
LV mass (g), mean (SD)	693	189.9 (66.6)	204.3 (72.0)	178.5 (59.7)	**<0.001**
LV mass index (g/m^2), mean (SD)		117.4 (39.5)	120.6 (40.2)	114.8 (38.8)	0.057
LVIDd (mm), mean (SD)		47.7 (7.2)	49.5 (7.7)	46.3 (6.5)	**<0.001**
LVIDs (mm), mean (SD)		31.7 (8.3)	33.5 (9.1)	30.3 (7.3)	**<0.001**
IVSs (mm), mean (SD)		10.8 (2.7)	10.9 (2.8)	10.7 (2.7)	0.537
IVSd (mm), mean (SD)		14.7 (3.2)	15.0 (3.2)	14.5 (3.1)	0.054
LVPWd (mm), mean (SD)		10.5 (2.0)	10.6 (2.1)	10.4 (1.9)	0.387
LVPWs (mm), mean (SD)		14.8 (2.7)	15.0 (2.8)	14.6 (2.6)	0.091
LVEDV (ml), mean (SD)		109.6 (39.9)	119.4 (44.8)	101.9 (33.7)	**<0.001**
LVEDV index (ml/m^2), mean (SD)		68.0 (24.4)	69.9 (26.1)	66.5 (22.8)	0.070
LVESV (ml), mean (SD)		44.6 (31.4)	51.1 (36.6)	39.5 (25.5)	**<0.001**
LVESV index (ml/m^2), mean (SD)		27.7 (19.1)	30.4 (21.7)	25.6 (16.5)	**<0.001**
LA volume (ml), mean (SD)	418	59.4 (24.5)	56.2 (23.2)	61.9 (25.2)	**0.019**
LA volume index (ml/m^2), mean (SD)		36.8 (16.0)	32.9 (13.6)	40.0 (17.0)	**<0.001**
LA area (cm^2), mean (SD)		19.9 (5.5)	19.2 (5.5)	20.4 (5.5)	**0.026**
LA area index (cm^2/m^2), mean (SD)		12.3 (3.7)	11.2 (3.2)	13.2 (3.8)	**<0.001**
EA, mean (SD)	584	1.1 (1.8)	1.0 (0.7)	1.1 (2.4)	0.630
Septal E/e’, mean (SD)	629	20.4 (12.5)	18.8 (11.1)	21.7 (13.3)	**0.003**
Lateral E/e’, mean (SD)	390	14.8 (7.8)	13.7 (8.4)	15.6 (7.2)	**0.016**
Average E/e’, mean (SD)	387	16.9 (8.4)	15.6 (9.1)	17.8 (7.7)	**0.012**
PASP (mmHg), mean (SD)	617	38.9 (15.0)	37.8 (14.9)	39.8 (15.0)	0.101

Abbreviations: Ao - aortic, AV - aortic valve, AVA - aortic valve area, IVS - interventricular septum, LA - left atrium, LV - left ventricular, EF - ejection fraction, LVH - left ventricular hypertrophy, LVID - left ventricular internal diameter, LVEDV - left ventricular end diastolic volume, LVESV - left ventricular end systolic volume, LVOT - left ventricular outflow tract, LVPW - left ventricular posterior wall diameter, MAC - mitral annulus calcification, MPG - mean pressure gradient, PASP - pulmonary artery systolic pressure, PPG - peak pressure gradient, RWMA - regional wall motion abnormality, RWT - relative wall thickness, SVi - stroke volume index, VTI - velocity time integral

Table S2. Echocardiographic parameters of aortic stenosis patients stratified by sex and severity (moderate and severe).

Variables	Moderate AS				Severe AS			
	n	**Male** ***n* = 96**	**Female** ***n* = 110**	***p*-value**	***n***	**Male** ***n* = 54**	**Female** ***n* = 55**	***p*-value**
AVA (cm^2), mean (SD)	206	1.1 (0.3)	1.0 (0.3)	0.378	109	0.7 (0.2)	0.7 (0.2)	0.097
AVA index (cm^2/m^2), mean (SD)		0.6 (0.2)	0.6 (0.2)	0.476		0.4 (0.1)	0.4 (0.1)	0.811
MPG (mmHg), mean (SD)		28.4 (7.9)	26.9 (5.6)	0.107		55.6 (14.7)	56.2 (14.5)	0.831
PPG (mmHg), mean (SD)		47.9 (16.3)	46.7 (16.8)	0.612		80.9 (22.3)	83.8 (26.8)	0.533
Vmax (m/sec), mean (SD)		337.1 (53.1)	327.9 (62.8)	0.279		439.7 (69.5)	449.4 (80.3)	0.515
Stroke volume (ml), mean (SD)		69.7 (22.5)	65.8 (20.4)	0.195		68.6 (26.3)	63.7 (19.9)	0.281
Stroke volume index (ml/m^2), mean (SD)		41.0 (13.1)	41.1 (12.1)	0.986		40.2 (14.4)	41.6 (12.1)	0.586
DI, mean (SD)		0.3 (0.1)	0.3 (0.1)	**0.015**		0.2 (0.1)	0.2 (0.1)	0.179
LVOT diameter (mm), mean (SD)		21.7 (2.1)	20.0 (1.6)	**<0.001**		21.4 (2.2)	20.3 (1.9)	**0.008**
LVOT VTI (mm), mean (SD)		20.9 (5.7)	23.2 (6.7)	**0.009**		20.7 (5.9)	23.3 (6.5)	**0.032**
LVOT Vmax (m/sec), mean (SD)		94.5 (20.6)	103.4 (26.2)	**0.008**		88.2 (21.0)	97.6 (25.0)	**0.036**
LVEF (%), mean (SD)		54.9 (15.4)	60.6 (12.3)	**0.004**		52.1 (14.4)	56.1 (12.9)	0.137
RWMA, *n* (%)	114	20 (33.9)	4 (7.3)	**<0.001**	20	4 (36.4)	3 (33.3)	>0.999
LV mass (g), mean (SD)	206	216.4 (68.5)	190.1 (66.0)	**0.006**	109	244.1 (80.4)	214.8 (60.3)	**0.035**
LV mass index (g/m^2), mean (SD)		127.0 (37.4)	118.8 (40.8)	0.141		143.7 (47.3)	140.4 (39.0)	0.697
LVIDd (mm), mean (SD)		50.4 (8.2)	46.9 (7.0)	**0.001**		49.9 (7.9)	47.5 (6.3)	0.093
LVIDs (mm), mean (SD)		34.4 (10.0)	30.2 (7.9)	**<0.001**		34.2 (8.8)	31.9 (7.3)	0.143
IVSs (mm), mean (SD)		10.9 (3.1)	10.4 (2.9)	0.297		10.8 (3.3)	10.4 (1.8)	0.375
IVSd (mm), mean (SD)		15.1 (3.1)	15.0 (3.1)	0.817		16.4 (3.1)	15.7 (2.9)	0.227
LVPWd (mm), mean (SD)		10.8 (1.7)	10.8 (1.6)	0.910		12.0 (2.5)	11.7 (2.3)	0.527
LVPWs (mm), mean (SD)		15.1 (2.6)	14.8 (2.4)	0.454		16.4 (3.0)	16.1 (2.9)	0.604
LVEDV (ml), mean (SD)		125.6 (47.9)	105.2 (37.5)	**<0.001**		121.6 (44.9)	107.6 (35.0)	0.075
LVEDV index (ml/m^2), mean (SD)		72.1 (27.3)	66.8 (24.5)	0.143		69.8 (24.0)	71.6 (25.0)	0.708
LVESV (ml), mean (SD)		55.2 (42.1)	39.5 (29.7)	**0.002**		52.9 (34.5)	43.9 (26.7)	0.130
LVESV index (ml/m^2), mean (SD)		32.5 (24.2)	24.8 (18.6)	**0.012**		31.1 (20.2)	28.6 (16.8)	0.492
LA volume (ml), mean (SD)	112	61.8 (27.5)	63.0 (20.4)	0.789	19	56.3 (24.6)	52.2 (20.2)	0.702
LA volume index (ml/m^2), mean (SD)		35.2 (15.6)	39.4 (12.9)	0.125		31.1 (12.6)	31.3 (11.2)	0.973
LA area (cm^2), mean (SD)		20.4 (6.3)	20.7 (4.4)	0.785		19.4 (5.4)	18.4 (4.8)	0.686
LA area index (cm^2/m^2), mean (SD)		11.7 (3.5)	13.0 (2.9)	**0.034**		10.7 (2.9)	11.1 (2.6)	0.790
EA, mean (SD)	177	1.0 (0.6)	0.9 (0.5)	0.209	93	0.9 (0.4)	1.1 (0.5)	**0.044**
Septal E/e', mean (SD)	187	20.0 (12.2)	21.2 (12.5)	0.512	100	22.1 (15.0)	24.5 (16.4)	0.461
Lateral E/e', mean (SD)	106	15.8 (10.3)	14.8 (6.8)	0.578	17	10.9 (3.0)	20.0 (7.1)	**0.002**
Average E/e', mean (SD)	106	18.2 (11.8)	17.2 (7.3)	0.595	17	13.8 (6.0)	19.4 (6.3)	0.084
PASP (mmHg), mean (SD)	188	38.4 (16.2)	36.7 (13.9)	0.449	99	38.0 (16.4)	42.4 (16.6)	0.183

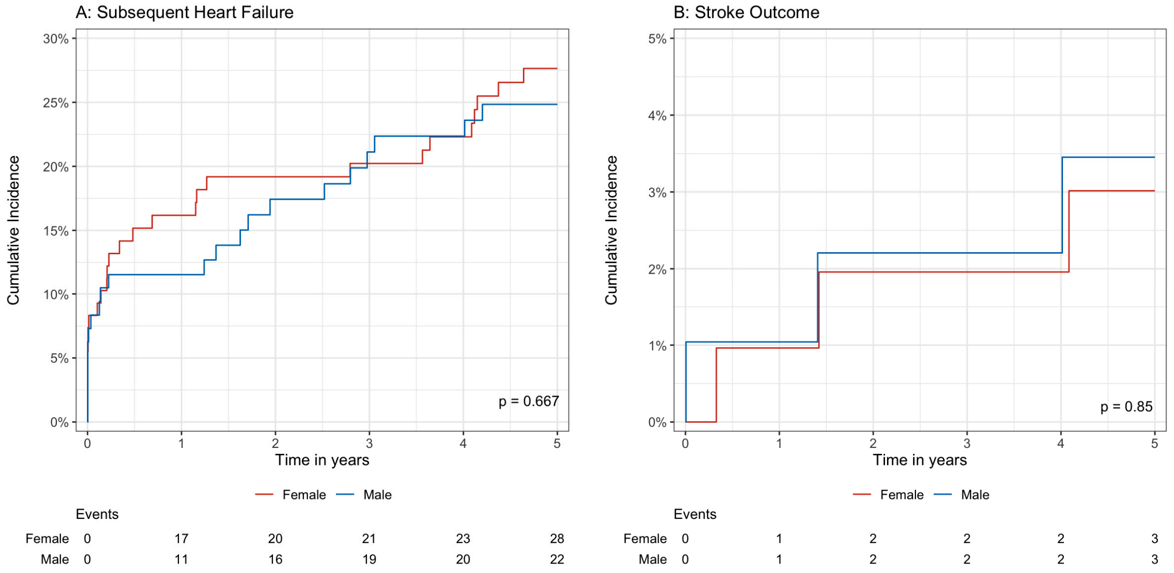

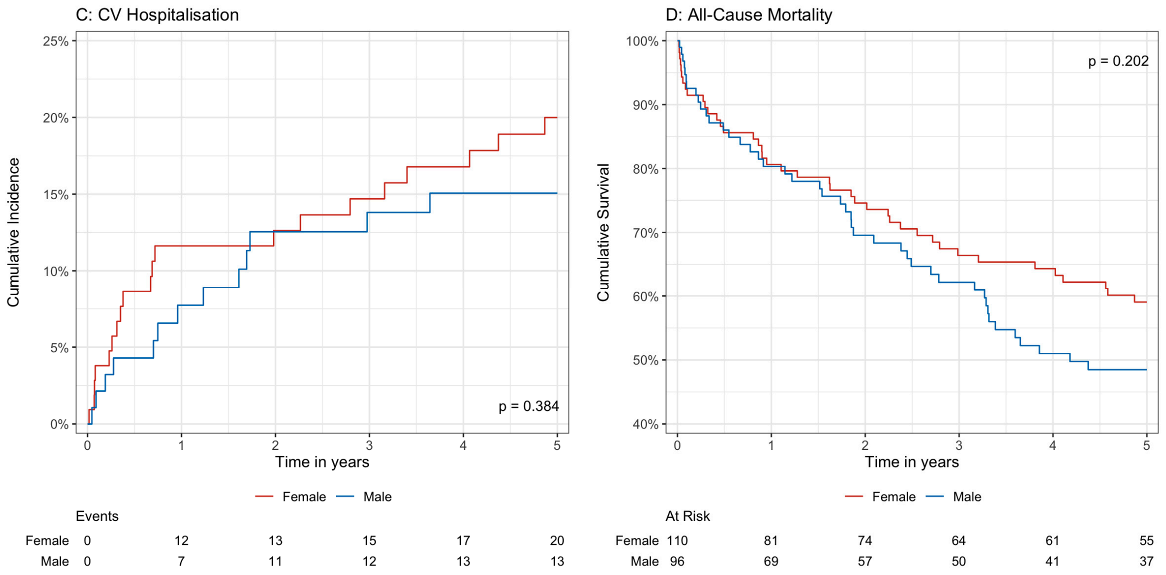

Figure S1(A-D). Kaplan-Meier and Cumulative Incidence Function estimates of outcomes comparing male and female moderate aortic stenosis patients

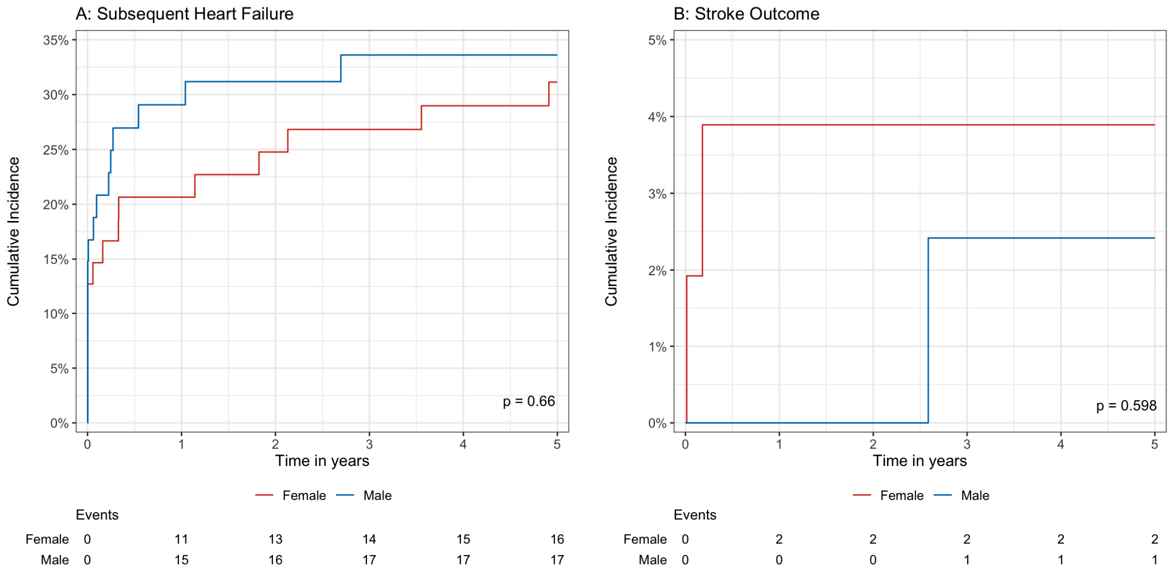

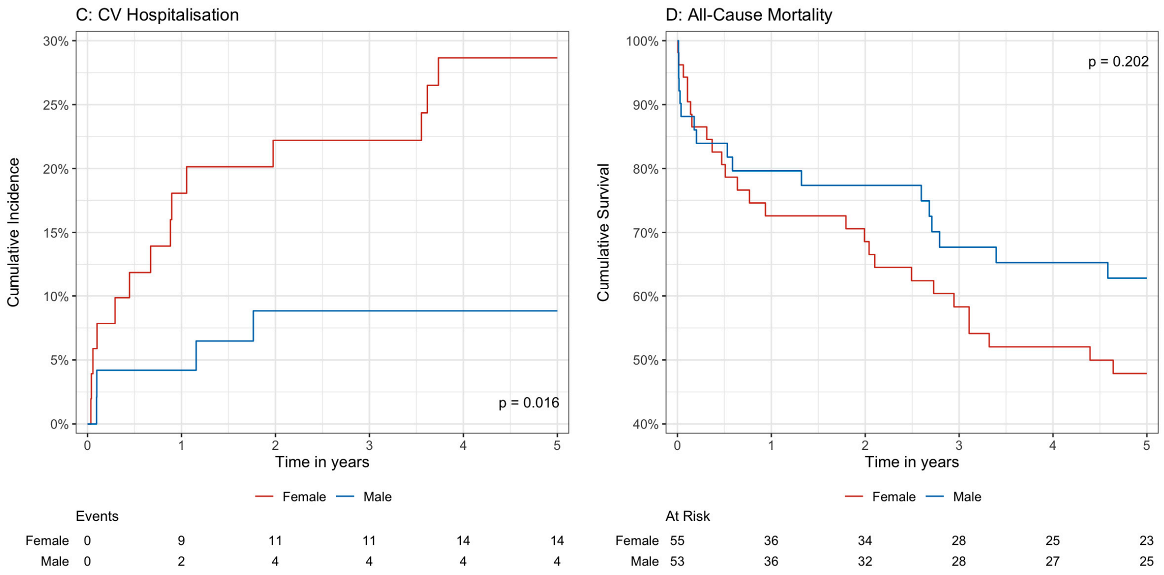

Figure S2(A-D). Kaplan-Meier and Cumulative Incidence Function estimates of outcomes comparing male and female severe aortic stenosis patients
